# Consistent timelines, divergent end points: plant community change in multiple tallgrass nitrogen addition experiments

**DOI:** 10.1007/s00442-025-05819-9

**Published:** 2025-10-28

**Authors:** Kimberly J. Komatsu, Meghan L. Avolio, John Blair, Sally E. Koerner, Zak Ratajczak, Melinda D. Smith, Ellen Welti, Kevin R. Wilcox, Lydia H. Zeglin

**Affiliations:** 1 Department of Biology, University of North Carolina Greensboro, Greensboro, NC USA; 2 Department of Earth and Planetary Sciences, Johns Hopkins University, Baltimore, MD USA; 3 Division of Biology, Kansas State University, Manhattan, KS USA; 4 Department of Biology, Colorado State University, Fort Collins, CO USA; 5 Great Plains Science Program, Smithsonian’s National Zoo and Conservation Biology Institute, Bozeman, MT USA

**Keywords:** Mesic grassland, Fertilization, Eutrophication, Global change, Community composition, Nitrogen

## Abstract

Human activities have increased nitrogen (N) bioavailability broadly, resulting in increased plant productivity and decreased plant species richness globally. However, the determinants of the rates of change and the ultimate plant community composition resulting from N additions remain unclear. Here we synthesize data from six long-term N addition experiments in a tallgrass prairie ecosystem to (1) determine whether plant compositional change with N additions occurs gradually or is an abrupt response, (2) identify short-term (*i.e.*, pulse) environmental drivers that may cause compositional changes, and (3) map the end points of plant communities following a decade or more of N addition. We find that most plant community change occurred gradually over the first several years of chronic N additions, with no evidence that other perturbations (*i.e.*, precipitation, herbivory, fire) catalyzed the change. Rather, we think that the observed pattern reflects the long-lived nature of dominant plant species within this community. Additionally, we find that the final community composition differed across the six experiments we synthesized, seemingly driven by differences in the initial plant community composition. Despite different species coming to dominate the six experiments with N addition, these species shared functional identities as fast-growing, weedy species. The shifts in tallgrass plant community composition observed with increased N availability across the six experiments synthesized here may have consequences for the stability of ecosystem function in a world with altered N bioavailability. Long-term experiments are necessary to capture these responses to N addition, with short-term experiments likely underestimating the community responses to altered N.

**Supplementary Information:**

The online version contains supplementary material available at 10.1007/s00442-025-05819-9.

## Introduction

The increase in global bioavailability of nitrogen (N) in recent centuries has dramatically affected ecosystem structure and function worldwide (Ackerman et al. 2019; Gruber and Galloway 2008; Steffen et al. 2018; Vitousek et al. 1997). N is a critical element in all proteins, with the availability of N both limiting the activity, growth, and reproduction of organisms and often shifting the competitive dominance of plant species with different N use tradeoffs (Craine et al. 2002; Wedin and Tilman 1993; You et al. 2017). The consequences of N addition for plant communities are well-studied, including the general tendencies to increase primary production and decrease plant community diversity (Gough et al. 2000; Seabloom et al. 2021; Shi et al. 2024; Xia and Wan 2008). However, questions remain about what factors control rates of change and the ultimate outcomes of chronic N addition on plant communities (Komatsu et al. 2019; Muehleisen et al. 2023; Smith et al. 2015). Specifically, does the chronic (*i.e.*, press) nature of N addition lead to gradual shifts in plant community composition, or does this press interact with pulse events in the environment to trigger rapid rates of change? This question is particularly important given the increasing co-occurrence of anthropogenically altered environmental changes in this century (Easterling 2000; Richardson et al. 2023; Rillig et al. 2019; Steffen et al. 2018).

It is well established that N shifts plant community composition compared to locations not receiving additions. Chronic N addition may shift species abundances due to the physiological responses of extant species in a community (*i.e.*, favoring nitrophilic species at the expense of others) (Smith et al. 2009), resulting in a similar assemblage of species with different species abundances (*e.g.*, Collins et al. 2008). As N addition continues, novel species favored by high N conditions may immigrate into the community at the expense of other species, resulting in species turnover (*e.g.*, Xu et al. 2012). Shifts in species abundances (*i.e.*, rank differences), turnover of species (*i.e.*, changes in species identities), and the loss and gain of species all underlie changes in plant communities with chronic N addition (Avolio et al. 2019, 2022; Komatsu et al. 2019; Muehleisen et al. 2023; Wilfahrt et al. 2023). Yet, the timeline of these changes with chronic N addition, including potential triggers of each component of community change, remains unknown.

Plant community and ecosystem dynamics in grasslands are often mediated by the availability of co-limiting resources, including water, N, and light (Blair 1997; Seastedt and Knapp 1993). As a result, responses to chronic augmentation of N may be constrained by the availability of another resource (*e.g.*, water). Precipitation is an especially important determinant of plant responses to N enrichment, with global meta-analyses of N enrichment experiments across ecosystem types demonstrating a positive relationship between mean annual precipitation (MAP) and both the magnitude of increase in plant biomass (Xia and Wan 2008) and the decline in individual species abundances (Midolo et al. 2019) under elevated N. Less is known about how precipitation or soil water availability affects the timeline of plant community responses to long-term N enrichment in grasslands. The high interannual variability in precipitation that is characteristic of many grasslands (Knapp and Smith 2001) may constrain plant responses to added N, such that plant responses to N are minimal in years with low rainfall or enhanced in years with high rainfall (Ladwig et al. 2012). These year-to-year differences in the relative limitations of water and N could create triggers that alter when and how plant communities change in response to chronic N enrichment.

Herbivory is another key ecosystem process in grasslands, ensuring the cycling of energy and nutrients. Up to 50% of grassland primary production is consumed by herbivores (Cebrian and Lartigue 2004; Detling 1988), ultimately impacting plant community composition and structure. Grasslands are known for their charismatic megafauna (*e.g.*, bison), who have strong impacts on plant composition (Koerner et al. 2018; Ratajczak et al. 2022). But the more inconspicuous insect and small mammal herbivores also play a large role (Agrawal and Maron 2022; Denyer et al. 2007; Gibson et al. 1990; Kaufman and Kaufman 1997; Schowalter 2022). Grasshoppers, for example, annually consume ~ 20% of available forage in some rangelands (Hewitt and Onsager 1983), with their impact varying with N- and precipitation-driven alterations in forage quantity and quality (Ebeling et al. 2018; Joern and Gaines 1990; La Pierre and Smith 2016). Similar patterns have been shown for small mammals, which frequently have boom and bust cycles driven by precipitation and disturbance dynamics (Batzli 1992; Bennison et al. 2018; Noble et al. 2019). The impacts of small mammals could be especially strong because they often consume plant propagules, resulting in demographic bottlenecks (Bricker et al. 2010; Dylewski et al. 2020; Schnurr et al. 2004) that vary over time as mammalian feeding preferences and demographics fluctuate (Hope et al. 2021). Plant community composition may therefore shift as herbivores consume specific plant species or life stages, altering germination success or providing competitive release (Bricker et al. 2010; La Pierre et al. 2015; Lucas et al. 2021). Thus, much like precipitation, a boom in herbivore population sizes and/or rates of herbivory may also constitute a pulse event that catalyzes plant community change with chronic N additions.

Here, we leverage data from six long-term N addition experiments, spanning 14 years in their start dates, established in a native tallgrass prairie ecosystem to assess (1) whether plant community shifts with N additions are gradual or rapid, (2) what environmental factors might serve as catalysts for plant community change, and (3) consistency in the end points of plant community change across replicate experiments following long-term chronic N addition. We specifically expected these plant community responses would occur due to the reordering of existing species present in the plots at the beginning of the experiments (Avolio et al. 2019; Smith et al. 2009). We predicted that high precipitation years (Sala et al. 2012) and high herbivore abundance (La Pierre and Smith 2016) would promote rapid shifts in plant community composition. Finally, we predicted that the final plant community state would differ across these six N addition experiments depending on the initial plant community composition.

## Methods

Data were collected at the Konza Prairie Biological Station in northeastern Kansas, USA, and obtained from the Konza Prairie LTER website (http://lter.konza.ksu.edu/) linked to the Environmental Data Initiative Data Portal. Data included species composition data from six experiments, Belowground Plots (BGP) Burned (Blair 2024), BGP Unburned (Blair 2024), Phosphorous Plots (PPlots; Avolio et al. 2023), Nutrient Network (NutNet; Komatsu and Smith 2023), Invertebrate Removals (Invert Removals; Komatsu et al. 2023), and Chronic Addition of Nitrogen Gradient Experiment (ChANGE). Details about and the associated publications for the six nutrient addition experiments included in this synthesis can be found in Table 1. Briefly, all experiments added 10 g/m^2^ N per year at the start of each growing season, with three experiments adding N in the form of ammonium nitrate (BGP Burned, BGP Unburned, PPlots) and three experiments adding N in the form of a polymer-coated time-release urea with a 60–90-day release time (NutNet, Invert Removals, ChANGE). Data from experiments with multiple rates of N addition or other interacting resource addition treatments (*e.g.*, phosphorus [P] or potassium [K] addition) were subset to include only the 10 g/m^2^ N addition treatments in isolation. The one exception is the Invert Removals experiment, where 10 g/m^2^ per year of N, P, and K were added.

**Table 1:** Summary of attributes and references for each of the six N addition experiments from Konza Prairie LTER that were used in our analyses

Experiment	Years (duration)	Watershed	Fire Regime	Topographic Position	Soil Type	N form	Reps	Plot size (Species Comp Subplot)	References
Belowground Plots (BGP) Burned	1986–2016 (31 yr)	HQ	Annual	lowland	Tully	NH_4_NO_3_	4	144 m^2^ (10 m^2^)	(Collins 1998)
Belowground Plots (BGP) Unburn	1986–2016 (31 yr)	HQ	Unburned	lowland	Tully	NH_4_NO_3_	4	144 m^2^ (10 m^2^)	(Collins 1998)
Phosphorus Plots (PPlots)	2003–2023 (21 yr)	2C	2 yr return	upland	Florence	NH_4_NO_3_	8	25 m^2^ (1 m^2^)	(Avolio et al. 2014)
Nutrient Network (NutNet)	2008–2023 (15 yr)	2C	2 yr return	upland	Florence	Urea	3	25 m^2^ (1 m^2^)	(La Pierre et al. 2016)
Invertebrate Removal (Invert)	2009–2018 (10 yr)	2C	2 yr return	upland	Florence	Urea	3	4 m^2^ (1 m^2^)	(La Pierre & Smith 2016)
ChANGE	2013–2023 (11 yr)	R1B	Annual	upland	Florence	Urea	6	25 m^2^ (1 m^2^)	Linabury et al. *in review*

Watershed indicates the location of each experiment within the Konza Prairie LTER

Plant species percent cover was estimated within permanent subplots nested within larger experimental plots in each experiment synthesized here (see Table 1 for plot and species composition subplot sizes). Within each subplot, plant species were identified and aerial cover estimated to the nearest 1% twice per growing season (May/June, August/September), with the exception of the BGP experiments, which were only sampled once every five years, starting in year 4 of nutrient addition treatments. Within each plot, the maximum cover value across the two sampling periods in a growing season was retained for each species to account for peak cover of early and late season species. Species overlap across all six experiments is relatively high, including dominance of grasses such as *Andropogon gerardii* and *Schizachyrium scoparium*, with a variety of subdominant and rare species making up most of the diversity of the plant communities.

Additional data related to potential drivers of plant community change were collected from the Environmental Data Initiative Data Portal, including precipitation (Nippert 2024), grasshopper populations (Joern 2023), and small mammal populations (Kaufman 2023). Precipitation and temperature data were collected from a weather station located at the Konza Prairie Biological Station headquarters (Nippert 2024), located within 13 km of all experiments included in this synthesis. From these climate data, growing season precipitation was calculated as the sum of rainfall from May through August each year. Yearly precipitation was calculated as the sum of rainfall over the water year that includes winter recharge and the growing season (September through August), rather than the calendar year (January through December) to account for the timing of biotic measurements. Grasshopper (Joern 2023) and small mammal (Kaufman 2023) abundances were obtained from watersheds that were not grazed by large ungulates and were burned either annually, every two years, four years, or twenty years (unburned). While the sample collection sites were not specifically co-located with the N addition experiments synthesized here, they span the landscape of the Konza Prairie Biological Station and therefore are representative of general herbivore abundances in each year at the site. Grasshopper and small mammal abundances were collected at peak abundance each year from multiple transects at each collection location and were averaged across transects first within each watershed, then by burn frequency, and finally across all burn frequencies to get one abundance value for each taxa per year at the site.

All data processing and analysis were performed in the R programming language version 4.1.3 (R Core Team 2024), with code archived at https://github.com/klapierre/konza-nutrient-synthesis. To determine how plant communities change over time with chronic N additions, plant species percent cover data were relativized by total cover within each plot and used to calculate Euclidean distances between the centroids of treatment and control plots in PCoA space based on Bray–Curtis dissimilarity using the multivariate_difference function in the *codyn* package (Avolio et al. 2019). Differences between treatment and control plots in multivariate space in each year (δY) were then compared through time by calculating yearly change in community difference as: δY_n_ – δY_n-1_. Additionally, we used the RAC_difference function in *codyn* to determine differences between each control plot and all possible N addition plots in each year for each experiment with respect to plant species richness, species identity, rank order, and evenness. Composition difference in each year between control and N added plots was then related to proportional richness differences, proportional species differences (*i.e.*, number of different species between control and N plots), rank differences, and evenness differences using linear multiple regression.

To assess the influence of naturally occurring pulse events on the timeline of plant community change, annual changes in community difference for each experiment were compared to the same year’s grasshopper and small mammal abundance and annual and growing season precipitation, as well as separate models comparing to the previous year’s grasshopper, small mammal, and precipitation data, using two-tailed Pearson correlation tests. Anomalies from the mean (absolute value of standardized abundances) of each variable were also compared to annual changes in community difference for each experiment; however, results were qualitatively similar to the analysis using raw data and therefore are not presented here. Finally, to determine the endpoints of compositional change with chronic N additions, the first and last year of plant community composition data for each experiment were plotted in an NMDS. Similar NMDS were generated to examine trajectories of community change through time for each experiment, which included all experiment years. Average species abundances within each experiment were compared over the first five years of experimental treatments for experiments with yearly data collection (PPlots, NutNet, Invert Removals, and ChANGE).

## Results

**Fig. 1:**
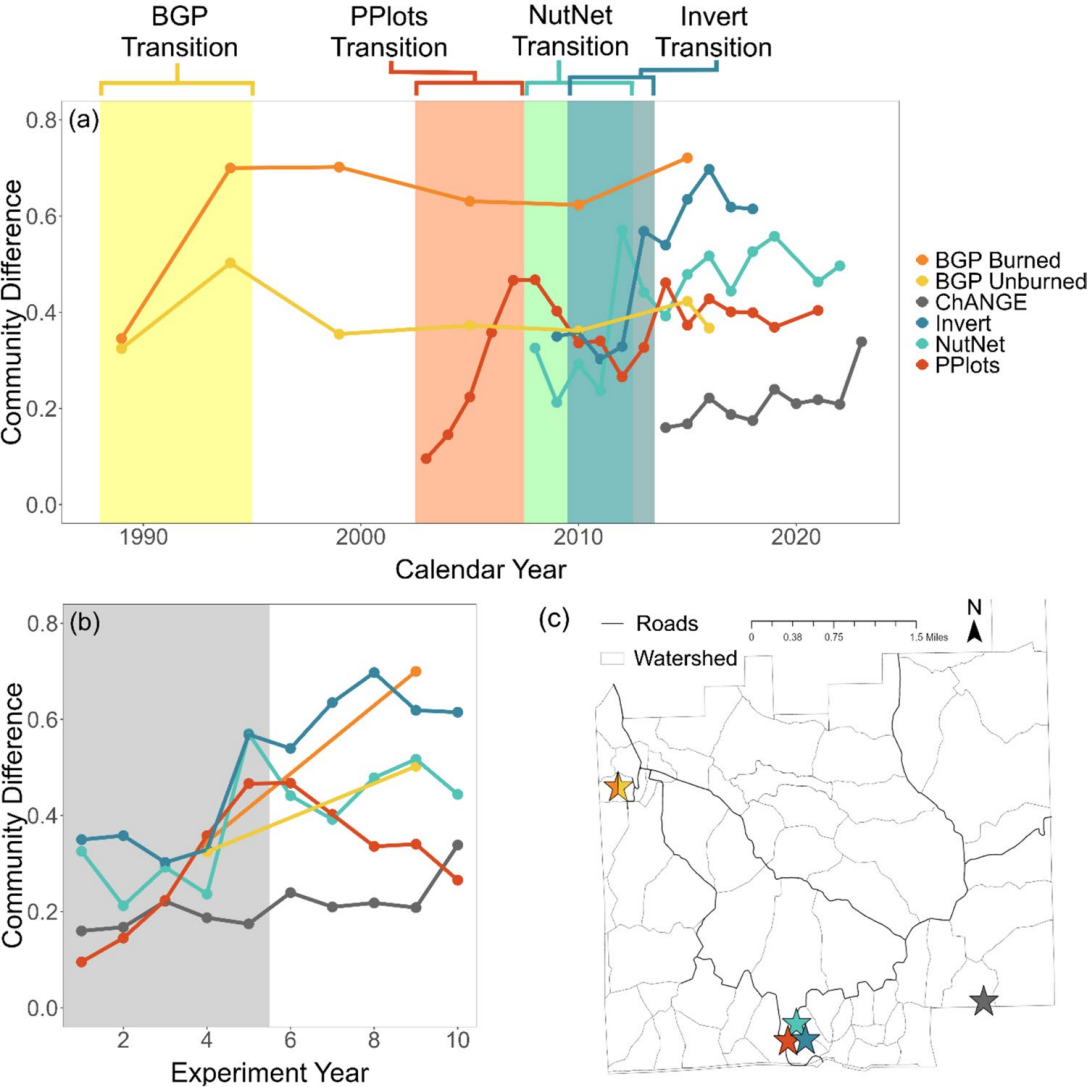
Temporal trajectory of difference in plant community composition between control and 10 g m^−2^ nitrogen added plots across six experiments at Konza Prairie Biological Station. (**a**) Responses of individual experiments through time, with key community transitions highlighted for the five experiments where change was observed. (**b**) Community transitions for all experiments overlayed by year of the experiment, with the shared key transition period across all experiments shown in grey. Note, species composition data from BGP experiments was only collected once every five years. (**c**) Map of Konza Prairie Biological Station, with the locations of each experiment shown as stars

Plant community differences between treatment and control plots increased over the first five years of N addition, and then remained elevated or oscillated around an elevated value for 3 of the 6 experiments examined (PPlots, NutNet, Invert Removals experiments; Figs. 1, S1, S2). Change occurring within the first five years of N addition is also plausible for the two BGP experiments, although sampling was not frequent enough to pinpoint the exact timeline (Figs. 1, S1, S2). This consistent timeline of divergence between control and N added plots occurred despite the experiments starting in different calendar years (Fig. 1a) and being located at different topoedaphic positions and burn management regimes.

**Fig. 2:**
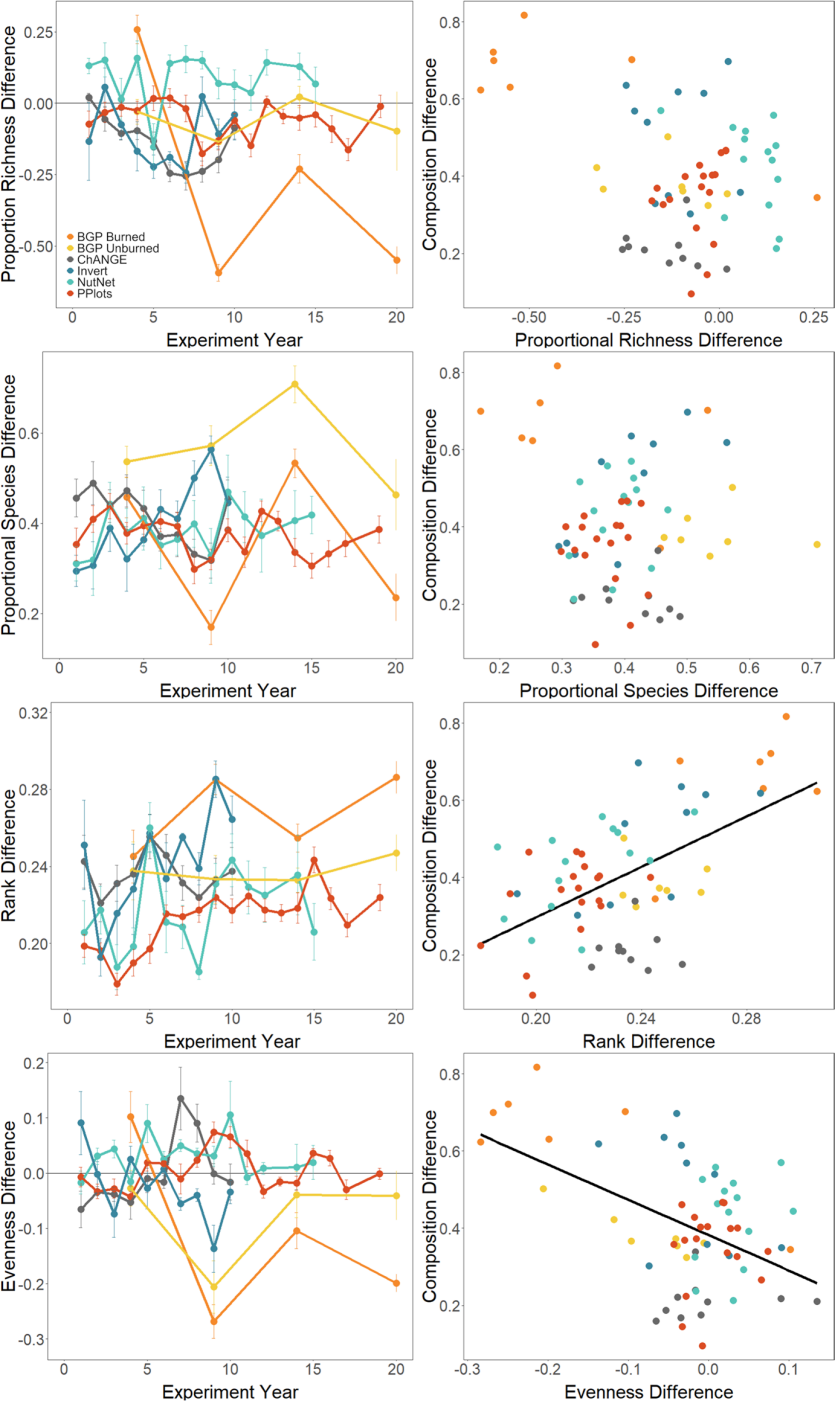
(left column) Proportional species richness and rank differences tend to increase over the first five years of N additions, while species and evenness differences do not show consistent temporal trends. Points and error bars indicate means and standard errors. (right column) Richness and rank differences significantly correlate with compositional difference. Point and line colors indicate the N addition experiment. Significant relationships are shown as solid black lines

**Fig. 3:**
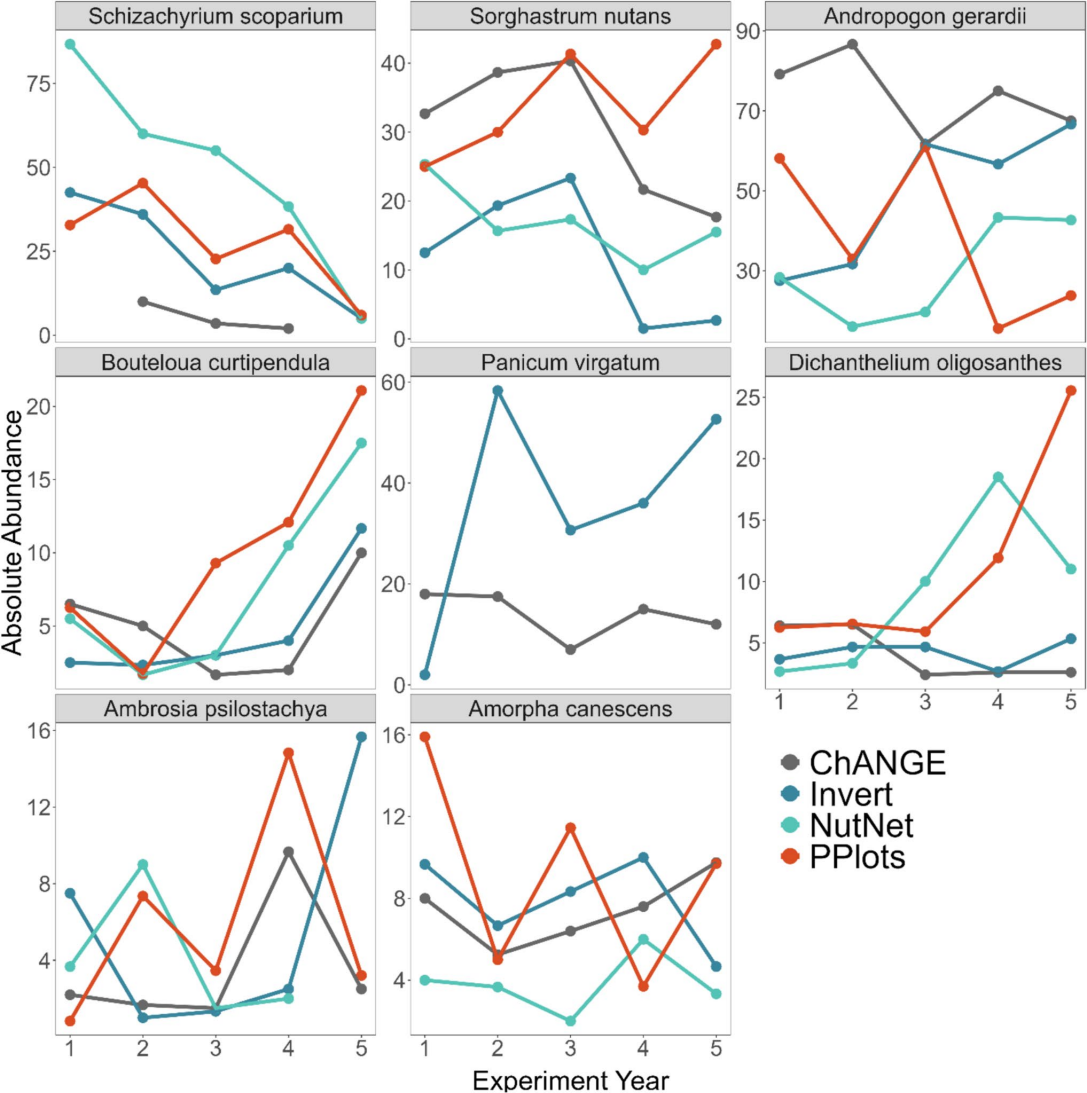
Individual species exhibit different temporal trajectories in absolute abundance (% cover) over the first five years of N additions. Point colors indicate the N addition experiment. Note the differing y-axis scaling across panels. BGP experiments are not shown due to infrequent community sampling

The multiple regression relating compositional difference to metrics of community difference (F_4,61_ = 10.06, p < 0.001, adj R^2^ = 0.358; Fig. 2; Table S3) demonstrated that the compositional difference between control and N added plots was significantly positively related to rank differences (p < 0.001) and negatively related to evenness differences (p = 0.009), marginally negatively related to species richness differences (p = 0.064), and not related to species differences (p = 0.137). Species re-ordering was apparent over the first five years of the experiments (Fig. 2), with some species increasing in abundance while others declined (Fig. 3). *Schizachyrium scoparium* (C_4_ grass) and *Amorpha canescens* (legume) consistently declined over time with N additions across all experiments (Fig. S4). In contrast, many species exhibited inconsistent responses across the N addition experiments examined. For example, *Andropogon gerardii* (C_4_ grass) more than doubled in abundance with N additions in NutNet and Invert Removals, while its abundance more than halved in PPlots. *Dichanthelium oligosanthes* (C_3_ grass) increased in abundance in PPlots, exhibited a transient increase in abundance in NutNet, and did not change in abundance in Invert Removals (Fig. 3, S4).

**Fig. 4:**
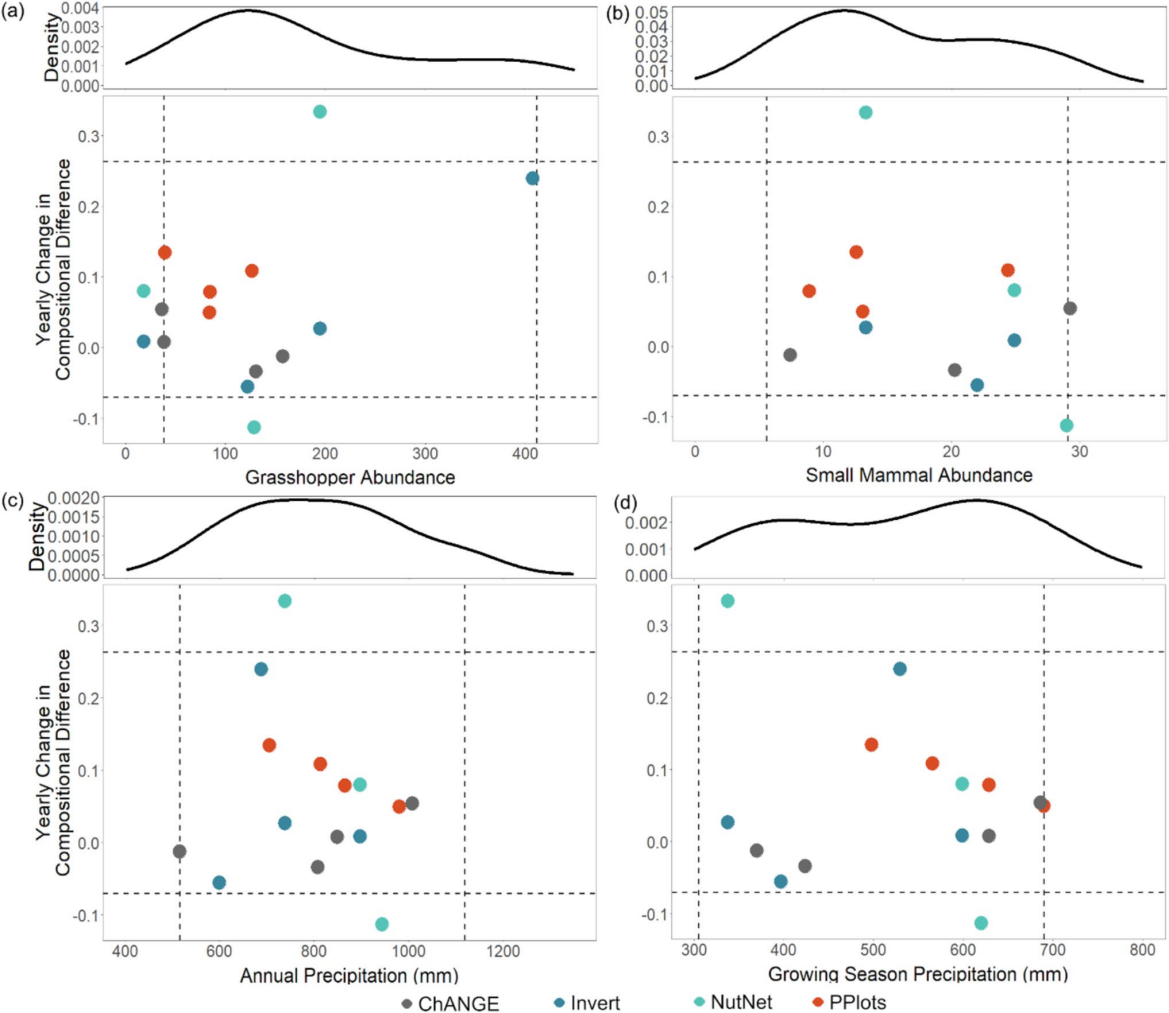
Yearly change in plant community compositional difference between control and N added plots during the first five years of treatment related to hypothesized drivers of change, including (**a**) grasshopper abundances, (**b**) small mammal abundances, (**c**) annual precipitation, and (**d**) growing season (May-Aug) precipitation. Shown are 95% quantiles for each driver and the community response (dashed lines), along with density plots of the historical distributions of each driver from 1983–2023. No significant correlations were observed between any of the drivers and the yearly change in compositional difference (p > 0.05 for all). Point colors indicate the different N addition experiments. Only four of the six experiments are depicted, as BGP community composition measurements were too infrequent (once every five years) to calculate yearly change. Small mammal data were not available for 2013–2015

Yearly change in the difference between control and N added plots for the first five years of each experiment was not significantly related to the same year’s or previous year’s grasshopper abundance, small mammal abundance, annual precipitation, or growing season precipitation (Fig. 4; Table S5). The addition of other limiting nutrients (P, K) or removal of invertebrate herbivores in combination with N additions did not alter temporal dynamics of plant compositional differences (Fig. S2), with temporal trends similar among treatments with N added alone or in combination with other limiting resources or the removal of invertebrate herbivores.

**Fig. 5:**
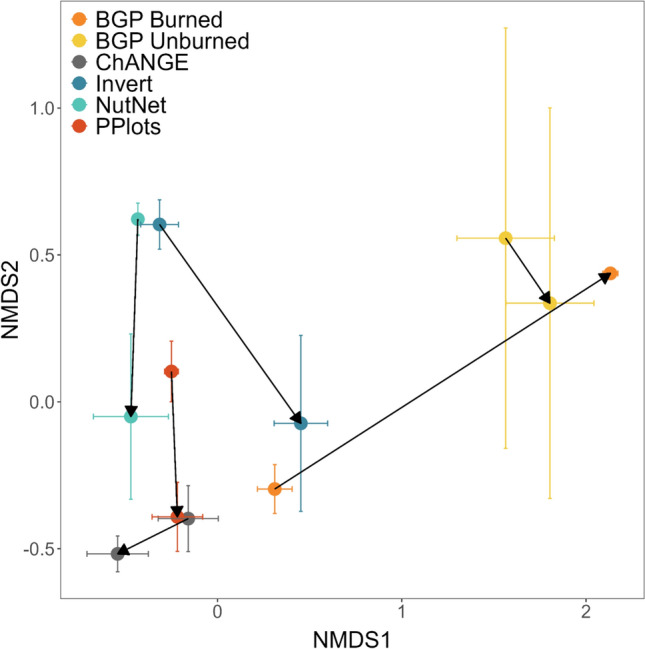
Ordination of plant community composition comparing control plots (arrow start) versus N addition plots (arrow end) in the final year of each of six experiments at Konza Prairie Biological Station. Nutrients added included N (10 gm^−2^ for all experiments) either alone or in combination with P and K (10 gm^−2^ each) in the Invert experiment only. Shown are means across all plots within each experimental treatment of each experiment ± standard errors

Ordination demonstrated that plant communities in each experiment’s final year differed between N addition and control plots; however, no consistent endpoint was apparent across all six experiments (Fig. 5). Similar ordinations through time demonstrate variable trajectories for each experiment following the initial community change, with some experimental communities oscillating around a new mean, while others began to revert to the control conditions (Fig. S5).

## Discussion

Here, we set out to assess (1) whether plant community shifts with N additions are gradual or rapid, (2) what environmental factors might serve as catalysts for plant community change, and (3) consistency in the end-points of plant community change across replicate experiments following long-term chronic N addition. Remarkably, despite two-fold variation in grasshopper abundance, small mammal abundance, and precipitation during times of plant community change across our six nutrient addition experiments, we found that plant communities steadily transitioned to new states during the first several years of N addition in each experiment, although exhibiting moderately different rates of divergence from control plots in any given experimental year across the experiments. We posit that this lengthy transition time is due to the time it takes for perennial species that dominate under lower N availability to be supplanted by high resource acquisition species, rather than any environmental trigger driving the response. Yet, these replacement communities are not consistent in species identity, despite similarities among experiments in topoedaphic conditions and burn histories, indicating that widespread N runoff into natural areas may lead to greater spatial variation in plant communities. Below, we explore these dynamics in additional detail.

The timelines over which differences in plant community composition became apparent between control and N addition plots were similar across the six experiments synthesized here, notable in both the metric of community difference from control plots and NMDS ordinations over time. Plant community divergence between control and N addition plots primarily occurred steadily across the first several years after the initiation of treatment application, followed by stabilization or oscillation around novel community states. This consistency in timelines suggests that our results are repeatable for this system (Schnitzer and Carson 2016), even across variation in topographies, fire regimes, precipitation, or herbivore abundances across the years of the experiment, and local plant communities. The one exception to this pattern was the ChANGE experiment, where N addition plots only showed high divergence from controls a decade after the experiment’s initiation. ChANGE is located in an annually burned upland, with plots having the highest abundance of the C_4_ grass *Andropogon gerardii* across the six experiments synthesized here. Because *A. gerardii* is adapted to variable conditions (Varvel et al. 2018; Yu et al. 2015), it may be able to persist under N addition pressure for longer than some of the other dominant species at the site, such as *Schizachyrium scoparium*. Alternatively, ChANGE is the most recently initiated experiment of the six synthesized here; therefore, it is possible that recent changes to the regional climate or nutrient aerosol deposition (Steffen et al. 2018) may have altered how the tallgrass plant communities at our site respond to N fertilization.

The plant community change we observed with N addition was gradual, rather than catalyzed by annual conditions in the year of change or the previous year. The lack of precipitation effects in mediating plant community differences was also unexpected, as this tallgrass prairie system is constrained by water availability (Jones et al. 2016). Additionally, we did not observe an effect of grasshopper and small mammal abundances on plant community divergence, contradicting the expectations that these consumers are often N limited (Le Roux et al. 2020; Mattson 1980) and affect plant community composition when at high densities (Lucas et al. 2021; Moorhead et al. 2017). It is possible that the densities of grasshoppers and small mammals never reached outbreak levels during the time periods examined here, reducing their potential impact on plant community transitions. While not mediated by precipitation or small consumers, the divergence in plant communities with N addition observed here was related to shifting abundances of common plant species, resulting in the eventual replacement of the initially dominant plant species in the communities.

Avolio et al. (2015) and (2022) suggest that species compositional differences can be better understood by comparing rank abundance curves (RACs). Although species were lost with N addition in the experiments synthesized here, these losses were marginal (hovering around zero in most cases) and did not result in large compositional differences. Species differences, a measure of species turnover that corrects for differences in richness (Avolio et al. 2019; Baselga 2010), were fairly invariant over time and not significantly related to compositional differences. Instead, we found that species re-ordering in the community (*i.e.*, rank differences) and differences in evenness drove the compositional response between control and N addition plots across experiments. The occurrence of rank differences without species differences demonstrates that the same species are generally found in all treatments, but the species who dominate differ among the treatments. Similar re-ordering has been observed in response to nutrient additions at other sites (Collins et al. 2008), and rank differences are often associated with compositional differences across experimental global change treatments (Avolio et al. 2022). We also found that evenness generally decreased with N addition, particularly in the lowland experiments, corresponding with a single species becoming more dominant. However, this response may not be consistent with those in other grassland types, as a synthesis of grassland nutrient addition experiments found no global trend in evenness responses to N (Seabloom et al. 2021).

The only consistently observed response across the six experiments synthesized here was the decline of the C_4_ grass *S. scoparium* with chronic N additions, regardless of its initial abundance in any given experiment. This response corresponds to previous research that indicates *S. scoparium* has a low R* for N, making it a strong competitor in low N environments but a weak competitor in high N environments (Craine et al. 2002; Wedin and Tilman 1993). The gradual decline of this perennial grass highlights the time that it takes for a dominant, long-lived species to lose its dominance with N additions, rather than exhibiting sudden shifts in response to other environmental pulse drivers.

Under elevated N conditions, we observed increased abundances of a few species that do not typically dominate under ambient conditions (rank and evenness differences); however, the identities of these new dominant species varied across the six experiments synthesized here, resulting in different endpoints of plant communities in NMDS ordination. These different endpoints of plant communities responding to chronic N conditions are surprising given the consistency of the timeline for plant community responses occurring over the first several years in all experiments. Some inconsistent species responses across the experiments include a doubling in the abundance of the C_4_ grass *Sorghastrum nutans* from 25 to 50% in PPlots, but exhibiting a halved abundance in NutNet (from 25 to 15%), Invert (from 10 to 3%), and ChANGE (from 30 to 18%). Similarly, another C_4_ grass, *Panicum virgatum*, increased in abundance from 2–3% in the Invert experimental plots to 40% abundance with chronic N additions. And while it was present in all experiments at the outset, the C_3_ grass *Dichanthelium oligosanthes* remained relatively low abundance in all but PPlots, where it increased to greater than 25% cover with chronic N additions.

While the topoedaphic conditions of each experiment may be expected to play a role in determining individual species responses to altered resource availability (Augusto et al. 2017; Castle and Neff 2009; Dong et al. 2023), this does not seem to be the case in our study. Some of the consistent species responses (*e.g.*, increased *P. virgatum*) occurred in experiments located in different soil types (deep and moist lowland Tully soils in BGP burned compared to shallow, rocky, and dry upland Florence soils in the Invert experiment). In contrast, some of the divergent species responses occurred in adjacent experiments (*e.g.*, increased *D. oligosanthes* in PPlots, but not NutNet and Invert experiments, which are co-located in the uplands of the same watershed). Rather, the initial pre-treatment abundances of the most responsive species appear to be a primary driver of the ultimate response. For example, *P. virgatum* appears to only have increased in the experiments where it was initially of low abundance (2–3% in Invert experiment), but not initially of higher abundance (~ 20% in ChANGE). *D. oligosanthes* only increased in the experiment where its initial abundance was slightly elevated compared to the others (6% initial abundance in PPlots, but 2–4% abundance across all other experiments). Thus, our results indicate that initial species abundances, more than abiotic conditions, may play the biggest role in determining the plant communities that result from chronic N additions. Similar trends also occurred in individual replicate plots within experiments, with winners with N additions relating to initial abundances (Koerner et al. 2016; La Pierre et al. 2015).

Often co-manipulated resources, rates of N addition, or form of N addition can influence grassland plant community and ecosystem responses to increasing N availability (Harpole et al. 2016; Peng et al. 2017; Tilman 1987). Across our six experiments, three had treatments that co-manipulated at least one additional resource (generally P). However, these multiple nutrient addition treatments did not show strong variation in the initial timeline of plant community response. Similarly, the N addition treatments compared across all six experiments utilized the same rate of N added (10 g/m^2^), preventing us from investigating directly the responses to the rate of N addition. A rate of N addition of 10 g/m^2^ is well above ambient rates of N and was implemented with the intention of relieving N limitation in the system, which likely drove the strong community responses we observed across our experiments. Aside from differences in topoedaphic conditions across our experiments, the primary experimental difference across experiments was the form of N added, with half of the experiments receiving N in the form of immediately available ammonium nitrate and the other half receiving time-release urea. The form of N added may have influenced the timeline of initial community divergence from control plots, which appeared to be lagged in NutNet and Invert Removals (urea), but gradual over the first several years in PPlots (ammonium nitrate). Unfortunately, the plant community sampling in the BGP experiments was not frequent enough for us to robustly test the differences in community response between immediate vs slow-release N fertilization. However, an offshoot experiment within the Nutrient Network has demonstrated similar plant richness and biomass responses to N added in the form of ammonium nitrate vs time-release urea at four sites (not including our study site; Borer et al. 2014). These patterns highlight the need for targeted comparisons to fully resolve how the rate and form of N addition, along with co-manipulated resources, may shape the pace and trajectory of community change.

While short-term experiments provide valuable information on immediate trajectories and magnitudes of response to global change drivers, long-term experiments provide insight into drivers of inflection points and ultimate outcomes of long-term change (Knapp et al. 2012; Komatsu et al. 2019; Reich et al. 2024). Although the inability to control stochastic ambient field conditions can be challenging, replicating experiments through time under different ambient conditions provides an exciting opportunity to unlock new insights. Established as one of the first LTER sites in 1981, Konza Prairie is uniquely poised in its 45th year to shed light on the context dependencies of long-term global change response trajectories (Franklin et al. 2016). Replicating experiments examining long-term plant responses to N addition under unique environmental conditions has allowed inference at a level that would not have been possible with fewer or shorter-duration experiments. As we are faced with the uncertain effects of projected global change, long-term experimental data with spatial and temporal replication are needed now more than ever to increase our understanding of interacting drivers and context dependencies of global change response trajectories.

## Supplementary Information

Below is the link to the electronic supplementary material.

Supplementary file1 (DOCX 467 KB)

## Data Availability

Data are published and publicly available: Blair 2024 (10.6073/pasta/2199a82aedbd8e462ac9edafd915d0fc); Avolio et al. 2023 (10.6073/pasta/4dd3761c45fcb9a05f1df600f20b76b7); Komatsu and Smith 2023 (10.6073/pasta/6e56960bb43fbcb29859c9e7178b7881); Komatsu et al. 2023 (10.6073/pasta/5f70e62694b1c95efc25385d01a2521c); Nippert 2024 (10.6073/pasta/910469efbf1f7e8d54c2b1ca864edec9); Joern 2023 (10.6073/pasta/e349433ba41c72fe98b6347ec9e7dd91); Kaufman 2023 (10.6073/pasta/d04abc9a9fe89a58e96d0764bee5bcf1). Statistical code is archived at https://github.com/klapierre/konza-nutrient-synthesis.
