# Supplementary material for: Consistent timelines, divergent end points: plant community change in multiple tallgrass nitrogen addition experiments
Source: Oecologia. 2025 Oct 28;207(11):178. doi: 10.1007/s00442-025-05819-9 (PMC12568805; doi:10.1007/s00442-025-05819-9)
Supplement: Supplementary file 1 — Supplementary file1 (DOCX 467 KB) [file 442_2025_5819_MOESM1_ESM.docx]

**Supplemental Information**


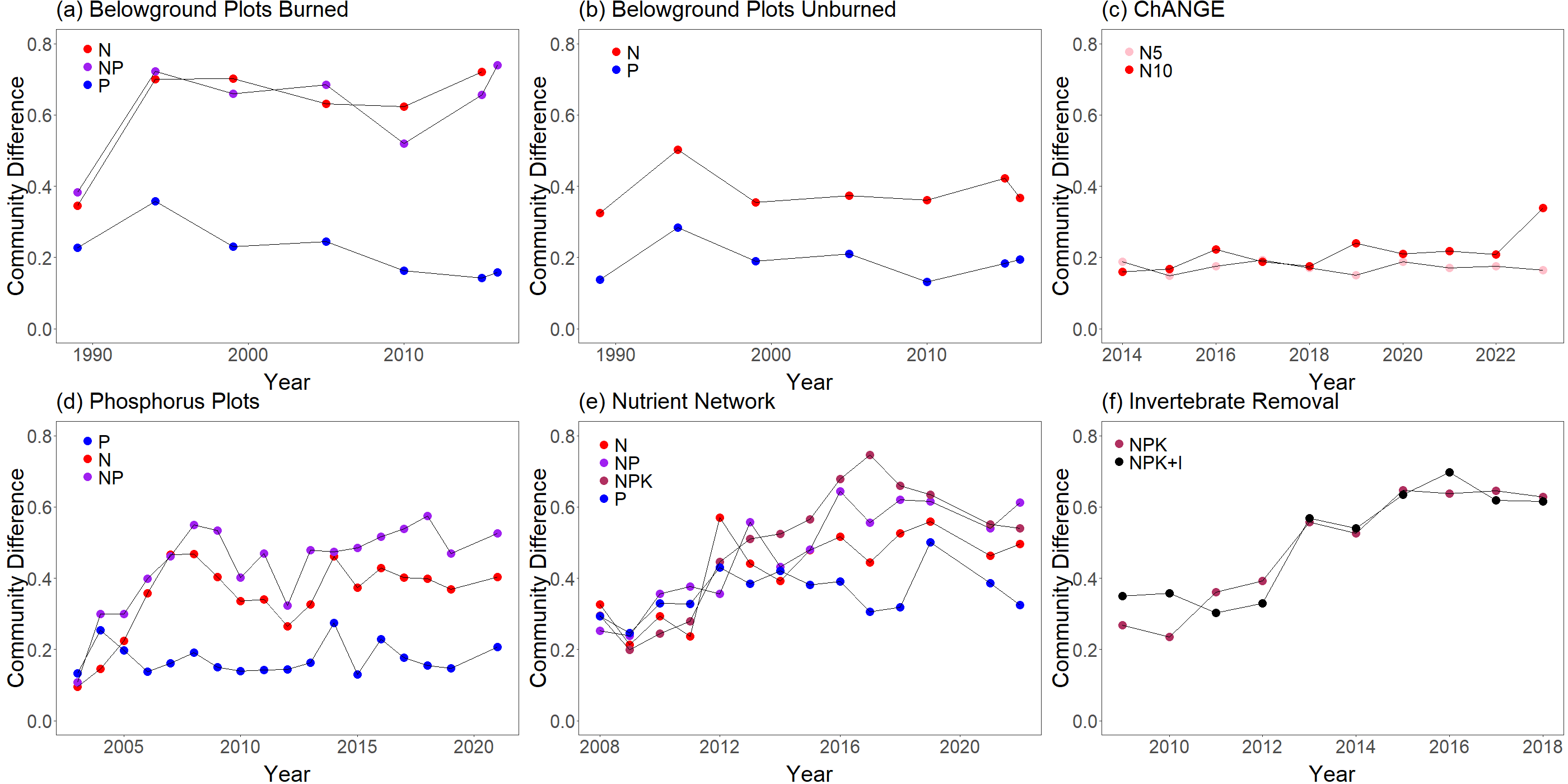


**Figure S1.** Temporal trajectory of difference in plant community composition between control and nutrient added plots across six experiments at Konza Prairie Biological Station. Nutrients added included N (10 gm^-2^ for all experiments, except ChANGE where amount added was either 5 or 10 gm^-2^) either alone or in combination with P (1 gm^-2^ in Belowground Plots, 10 gm^-2^ in all other experiments), K (10 gm^-2^ in Invertebrate Removal only), and/or insecticide (Invertebrate Removal only).


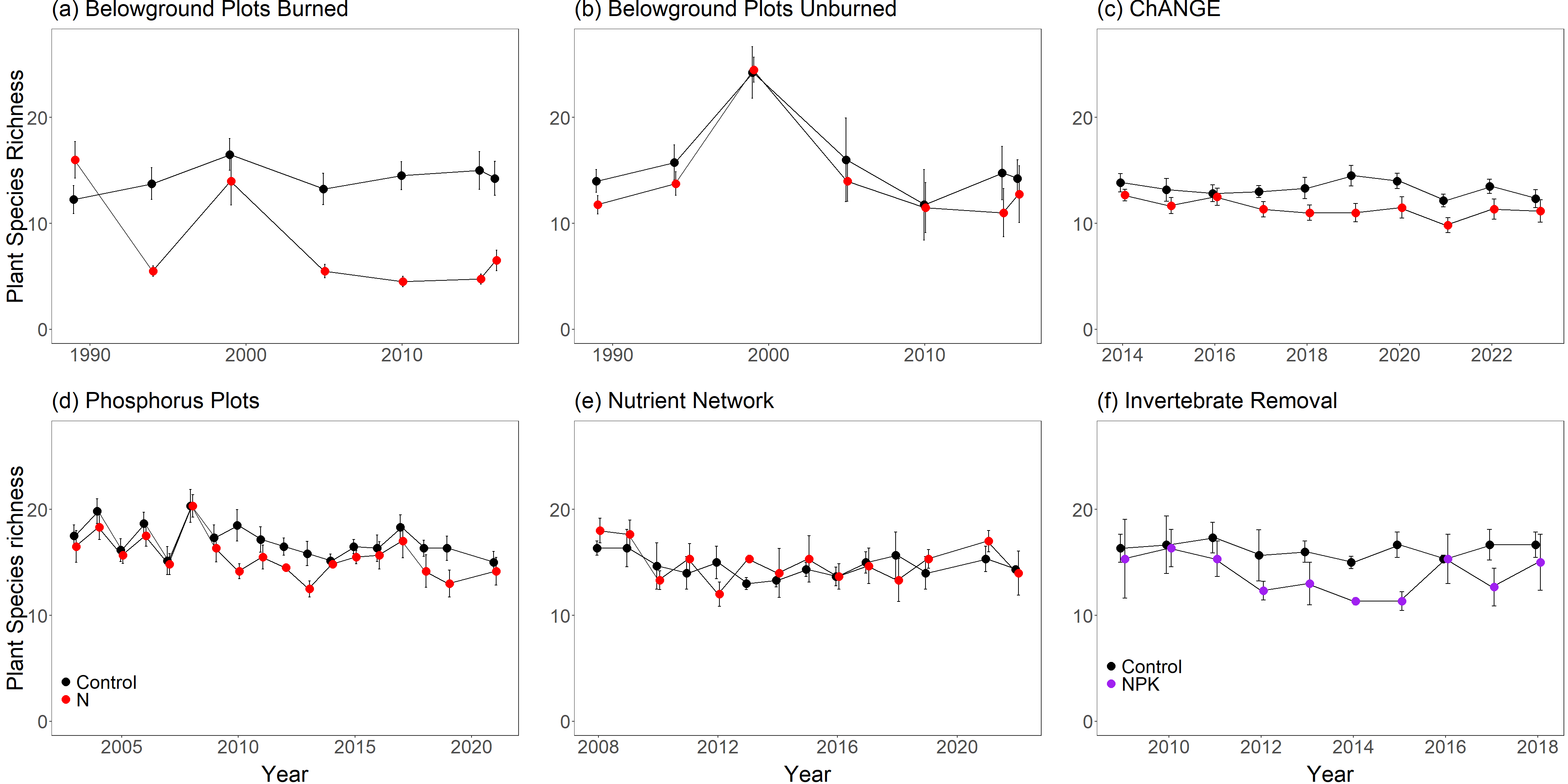


**Figure S2.** Temporal trajectory of plant species richness in control (black points) and N added (red points) plots across six experiments at Konza Prairie Biological Station. N was added at a rate of 10 gm^-2^ for all experiments. For the Invertebrate Removal plots, N was added in combination with P and K (10 gm^-2^ of each; purple points).

**Table S3.** The multiple regression relating plant compositional difference to metrics of community difference (overall model fit: F_4,61_=10.06, p<0.001, adj R^2^=0.358) demonstrated that the compositional difference between control and N added plots was significantly positively related to rank differences and negatively related to evenness differences, marginally negatively related to species richness differences, and not related to species differences.

|  | Estimate | Std. Error | t-value | p-value |
| --- | --- | --- | --- | --- |
| Intercept | -0.240 | 0.189 | -1.27 | 0.208 |
| Richness Difference | 0.286 | 0.151 | 1.89 | 0.064 |
| Species Difference | -0.302 | 0.201 | -1.51 | 0.137 |
| **Rank Difference** | **3.331** | **0.853** | **3.90** | **<0.001** |
| **Evenness Difference** | **-0.694** | **0.259** | **-2.68** | **0.009** |


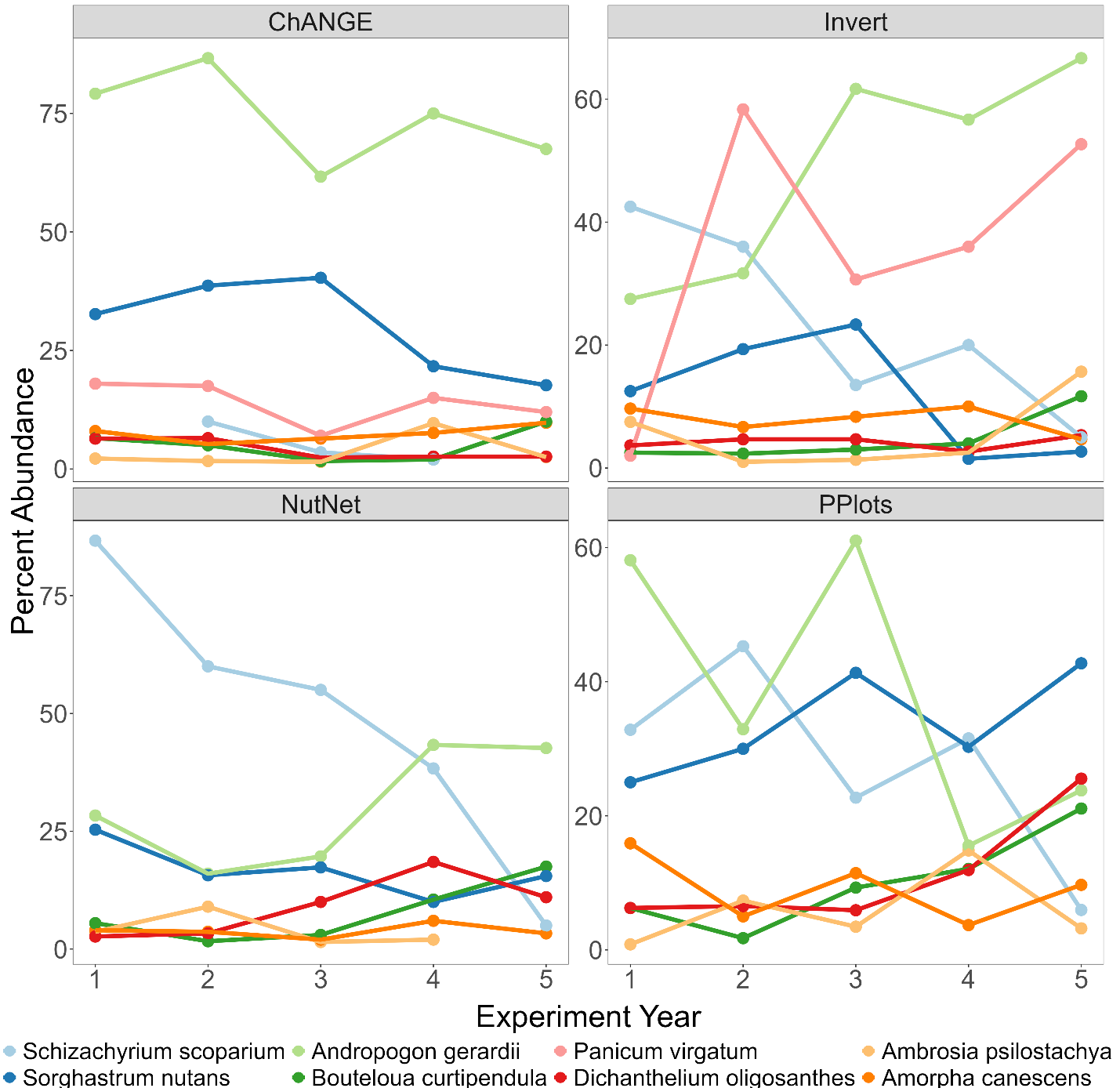


**Figure S4.** Temporal trends of the absolute abundances of individual species over the first five years of N additions differ across four N addition experiments. Point colors indicate the plant species. Note the differing y-axis scaling across panels. Belowground Plots experiments are not shown due to infrequent sampling timeline.

**Table S5.** Yearly change in the difference between control and N added plots for the first five years of each experiment was not significantly related to the same year’s or previous year’s grasshopper abundance, small mammal abundance, annual precipitation, or growing season precipitation.

| Lag |  | t-value | df | p-value | r |
| --- | --- | --- | --- | --- | --- |
|  |  |  |  |  |  |
| Current Year | Grasshopper Abundance | 1.64 | 14 | 0.123 | 0.401 |
|  | Small Mammal Abundance | -1.30 | 12 | 0.217 | 0.352 |
|  | Annual Precipitation | -0.13 | 14 | 0.899 | -0.034 |
|  | Growing Season Precipitation | -0.34 | 14 | 0.739 | -0.091 |
|  |  |  |  |  |  |
| Previous Year | Grasshopper Abundance | 1.30 | 14 | 0.213 | 0.329 |
|  | Small Mammal Abundance | 0.91 | 12 | 0.382 | 0.265 |
|  | Annual Precipitation | -0.60 | 14 | 0.558 | -0.159 |
|  | Growing Season Precipitation | -0.48 | 14 | 0.641 | 0.126 |
|  |  |  |  |  |  |


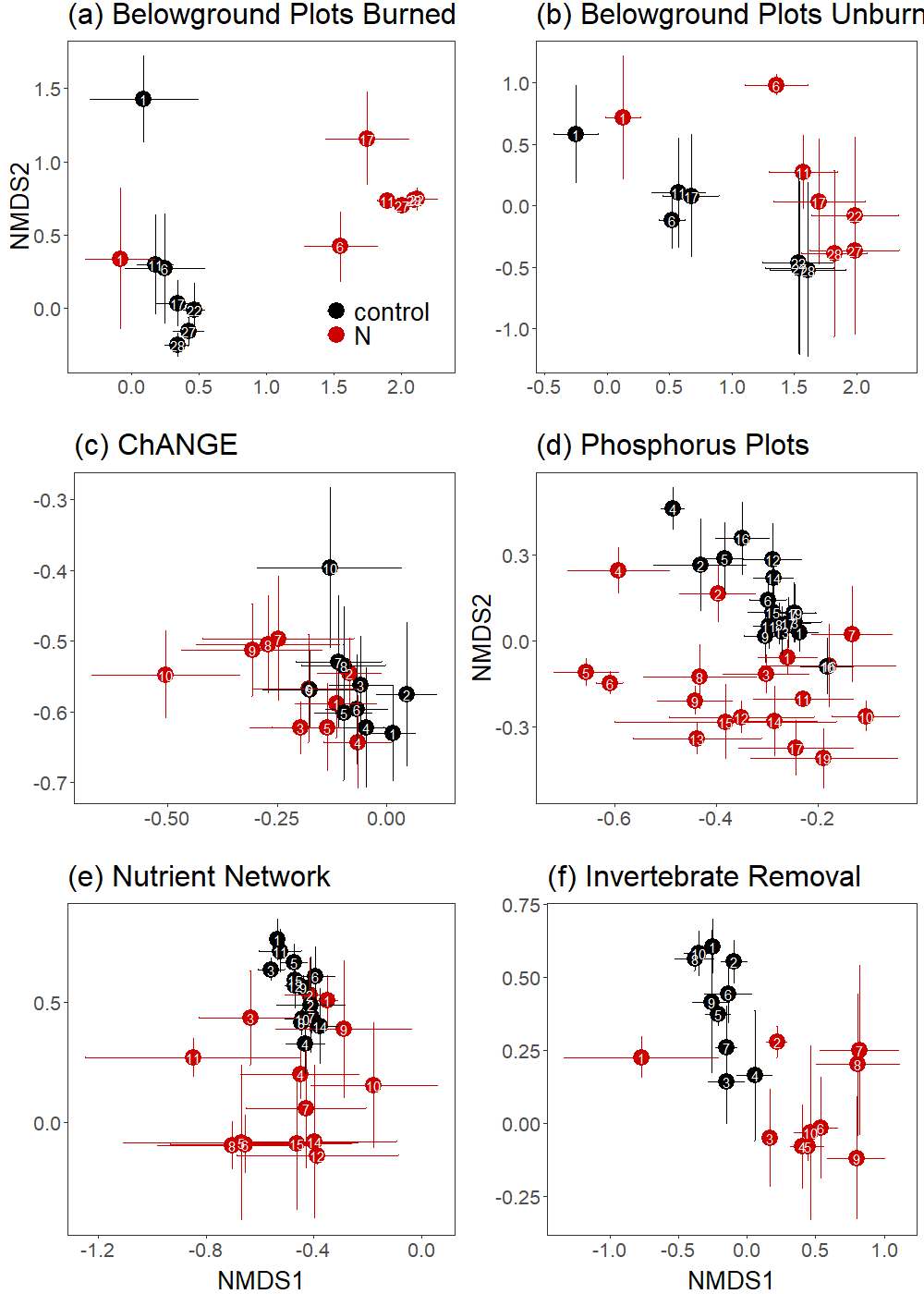


**Figure S5.** Ordination of plant community composition comparing control plots (black points) versus N addition plots (red points) across all experimental years (numbers) in each of six experiments at Konza Prairie Biological Station. Shown are means across all plots within each experimental treatment of each experiment ± standard errors.
